# The role of the immune system in posttraumatic stress disorder

**DOI:** 10.1038/s41398-022-02094-7

**Published:** 2022-08-04

**Authors:** Seyma Katrinli, Nayara C. S. Oliveira, Jennifer C. Felger, Vasiliki Michopoulos, Alicia K. Smith

**Affiliations:** 1 Department of Gynecology and Obstetrics, Emory University, Atlanta, GA USA; 2 National Institute of Woman, Child, and Adolescence Health Fernandes Figueira, Rio de Janeiro, RJ Brazil; 3 Department of Violence and Health Studies Jorge Careli, National School of Public Health, Fiocruz, Rio de Janeiro, RJ Brazil; 4 Department of Psychiatry and Behavioral Sciences, Emory University, Atlanta, GA USA; 5 The Winship Cancer Institute, Emory University, Atlanta, GA USA

**Keywords:** Psychiatric disorders, Predictive markers

## Abstract

Posttraumatic stress disorder (PTSD) develops in a subset of individuals upon exposure to traumatic stress. In addition to well-defined psychological and behavioral symptoms, some individuals with PTSD also exhibit elevated concentrations of inflammatory markers, including C-reactive protein, interleukin-6, and tumor necrosis factor-α. Moreover, PTSD is often co-morbid with immune-related conditions, such as cardiometabolic and autoimmune disorders. Numerous factors, including lifetime trauma burden, biological sex, genetic background, metabolic conditions, and gut microbiota, may contribute to inflammation in PTSD. Importantly, inflammation can influence neural circuits and neurotransmitter signaling in regions of the brain relevant to fear, anxiety, and emotion regulation. Given the link between PTSD and the immune system, current studies are underway to evaluate the efficacy of anti-inflammatory treatments in those with PTSD. Understanding the complex interactions between PTSD and the immune system is essential for future discovery of diagnostic and therapeutic tools.

## Introduction

Posttraumatic stress disorder (PTSD) is a debilitating psychiatric disorder characterized by re-experiencing of trauma, avoidance of trauma reminders, and hyperarousal symptoms that cause negative alterations in cognition, mood, and physiologic health [1]. PTSD is unique among other psychiatric disorders, as it requires trauma exposure to develop. Although over 70% of the population is exposed to at least one traumatic event during their lifespan, it is not clear why only some individuals develop PTSD [2]. Given the high comorbidities of inflammatory and metabolic disorders with PTSD [3–9], some studies have focused on the potential for an immune-related or inflammatory etiology for PTSD [10–12], whilst others suggest that PTSD promotes inflammation [13, 14] or that a bidirectional relation between PTSD and inflammation exists [15, 16].

In this review, we first summarize potential mechanisms connecting PTSD and the immune system. We describe studies of peripheral immune markers and mechanisms through which immune alterations affect neurotransmitter systems and brain regions that contribute to PTSD symptomatology. We also highlight the contribution of chronic inflammation in conditions often co-morbid with PTSD. Finally, we explore plausible therapeutic strategies targeting the immune system, based on its interaction with PTSD.

## PTSD and inflammation

**Fig. 1:**
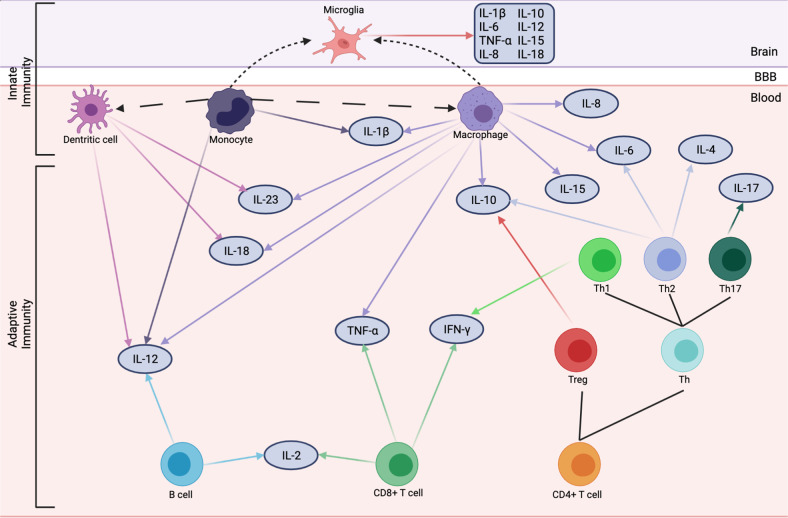
Immune cells and cytokines implicated in PTSD. Long dashed lines represent differentiation. Short dashed lines represent trafficking into the brain. BBB blood–brain barrier, IL interleukin, Th T helper cell, Treg regulatory T cell.

The neuroendocrine, psychophysiological, and neurobiological changes in PTSD etiology and outcome have been extensively studied [17, 18]. Growing evidence in the past two decades points to mechanisms related to the innate (i.e., non-specific first line of defense regulated by innate immune cells, including monocytes, macrophages, dendritic cells, and microglia) and adaptive (i.e., antigen-specific immunity regulated by T and B lymphocytes) immune systems in the pathophysiology of PTSD [17–19]. The initial evidence for the relationship between PTSD and the immune system comes from individual studies and subsequent meta-analyses reporting alterations in peripheral inflammatory markers, such as C-reactive protein (CRP), interferon-gamma (IFN-γ), interleukin-6 (IL-6), interleukin-10 (IL-10), and tumor necrosis factor-alpha (TNF-α) in individuals with PTSD (Fig. 1) [10, 20–23]. Moreover, hypothesis-free genome-wide [24, 25], epigenome-wide [26–30], and transcriptomic studies [31–35] of PTSD have identified multiple genes related to the immune system.

### Alterations in peripheral immune markers in PTSD

The inflammatory environment in PTSD is characterized by increased levels of pro-inflammatory markers (e.g., CRP, IL-6, IL-1β, IL-2, TNF-α, IFN-γ) and decreased levels of anti-inflammatory markers (e.g., IL-10) (Fig. 1) [10, 20–22]. Elevated inflammatory markers in PTSD may create a positive feedback loop to promote inflammation, such that IL-6, IL-1β, and TNF-α induce CRP to activate the complement system, which triggers a cascade of events to promote inflammation [18]. Nonetheless, it is still not clear whether the inflammatory milieu is the outcome of PTSD, or if pre-existing or trauma-induced inflammation increases the risk of PTSD. Notably, a bidirectional relationship between PTSD and inflammation is supported by recent reports [15, 16, 19], including a large-scale genetic study reporting a bidirectional genetic association between PTSD and CRP [16].

Longitudinal studies investigating whether PTSD development leads to inflammation reported that PTSD caused increases in inflammatory markers, including IL-1β, IL-8, CRP, and tumor necrosis factor receptor II (TNFRII) [36, 37]. On the contrary, Glaus et al. [38] observed lower IL-6 levels following PTSD diagnosis, indicating a decrease in inflammation. Longitudinal studies evaluating whether pre-existing inflammation is a risk factor for PTSD reported that increased levels of CRP and TNFRII predicted PTSD diagnosis [10, 15]. Transcriptomic studies conducted on US Marines revealed that upregulation of immune-related genes and overexpression of genes in networks associated with the innate immune response and interferon signaling at pre-deployment predicted post-deployment PTSD [39, 40]. Here, one can query the possible causes of pre-existing inflammation. Some possible drivers of this pre-existing inflammation (e.g., metabolic conditions, biological sex, and genetics) will be discussed below. Trauma and stress exposure across the lifespan might also contribute to inflammation prior to the incident trauma that results in PTSD [19]. Notably, a transdiagnostic meta-analysis of trauma exposure reported increased peripheral CRP, IL-1β, IL-6, and TNF-α concentrations in participants who experienced traumatic events (e.g., childhood maltreatment, natural disaster, violence) across their lifespan [41]. Specifically, early life adversity, including maltreatment, parental separation, and low socioeconomic status in childhood, is associated with increased CRP, IL-6, and TNF-α levels in adulthood [27, 42, 43]. Concordantly, studies that assessed inflammatory markers in the acute aftermath of trauma showed that increased levels of IL-6, IL-8, and CRP were associated with PTSD at follow-up [11, 12, 44]. In contrast, Michopoulos et al. [45] reported that decreased levels of TNF-α and IFN-γ upon trauma predicted chronic PTSD trajectory. However, since these studies measured inflammatory markers right after the trauma exposure, it is not clear whether the inflammatory response precedes the trauma or is the result of an acute posttraumatic response. The inability to assess the origin of the inflammatory response (i.e., pre- or post-trauma) may account for the inconsistent findings across the studies.

The relationship between inflammation and traumatic experiences is also supported by animal repeated social defeat stress (RSDS) models. For instance, IL-17A secreted from meningeal T cells in the brain was reported to control anxiety-like behavior in mice through neuronal IL-17a receptor subunit (IL-17Ra) signaling [46]. Following RSDS, anxiety-like behaviors are associated with increased levels of peripheral cytokines, including IL-2, IL-10, IL-17A, IL-22, and TNFα [47]. In contrast, Hodes et al. [48] showed increased peripheral cytokines in mice susceptible to social stress after RSDS.

### Link between the HPA axis, autonomic nervous system, and inflammation in PTSD

**Fig. 2:**
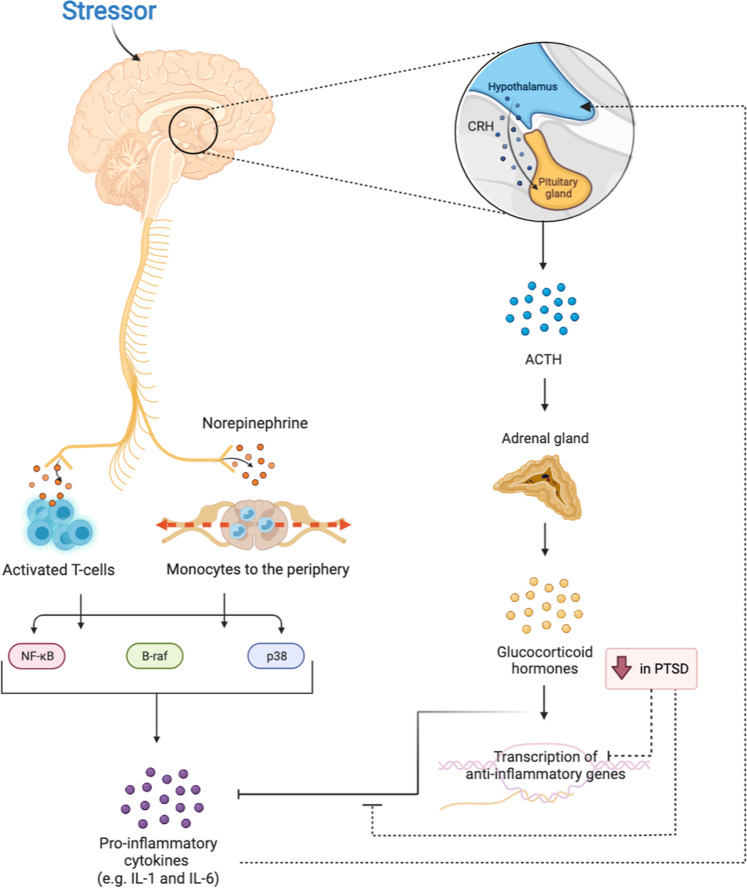
Relationship between the HPA axis, sympathetic nervous system, and inflammation in PTSD. Stress exposure stimulates sympathetic nervous system (SNS). Norepinephrine release from activated SNS fibers stimulates proinflammatory cytokines production through the NF-kB, B-Raf, and p38 pathways. The HPA axis is also activated upon exposure to stress, stimulating inflammatory responses that limit HPA reactivity. The reduced ability of glucocorticoids to inhibit inflammatory processes contributes to the proinflammatory environment in PTSD. CRH corticotrophin-releasing hormone, ACTH adrenocorticotropic hormone, NF-κB nuclear factor-κB, IL-1 Interleukin-1, IL-6 Interleukin-6.

The neuroendocrine stress response is comprised of the autonomic nervous system (ANS) and the hypothalamic–pituitary–adrenal (HPA) axis, which relay signals to the peripheral organs and the immune system (Fig. 2). Upon acute exposure to stress, corticotrophin-releasing hormone (CRH) is secreted from the hypothalamus, thereby activating the HPA axis [49]. The binding of CRH to its receptor on pituitary corticotropes triggers the release of adrenocorticotropic hormone (ACTH) from the anterior pituitary into the systemic circulation, stimulating glucocorticoid (cortisol in humans) synthesis from the adrenal cortex [49]. In parallel, stress exposure also triggers the sympathetic nervous system (SNS) to release catecholamines (e.g., epinephrine and norepinephrine), which are responsible for physiological changes, such as increases in heart rate and blood pressure [50]. In response to norepinephrine, monocytes are mobilized from the bone marrow into the periphery, where they encounter danger-associated molecular patterns (DAMPs), activating nuclear factor kappa B (NF-kB) mediated production of pro-inflammatory cytokines [51, 52]. Indeed, individuals with PTSD exhibited increased peripheral NF-κB activity and NF-κB-mediated transcriptional changes in monocytes, which contribute to the inflammatory environment [34, 35]. Similarly, norepinephrine release from activated SNS fibers further stimulates the NF-κB, B-raf-ERK1/2, and p38 pathways in activated T cells to produce pro-inflammatory cytokines [53–55]. Along with SNS activation, decreased parasympathetic activity in PTSD contributes to the inflammatory milieu [56]. Elevation in these pro-inflammatory cytokines, in turn, leads to HPA axis reactivity [17].

In PTSD, HPA axis hyperactivity following repeated trauma may disrupt glucocorticoid signaling, which leads to peripheral and central nervous system inflammation [17]. Normally, following the binding of glucocorticoids to the glucocorticoid receptor (GR), the stress response is dampened via a negative-feedback loop [18]. The glucocorticoid–GR complex also suppresses inflammatory responses either by stimulating the transcription of anti-inflammatory genes in the nucleus or by inhibiting the expression of proinflammatory proteins in the cytosol [18]. However, chronic exposure to stress may result in glucocorticoid resistance, wherein cortisol cannot inhibit NF-κB-mediated pro-inflammatory cytokine release, dampening glucocorticoid negative feedback of the HPA axis [17]. Overall, the inflammatory state in PTSD is propagated through a combination of glucocorticoid resistance along with increased sympathetic and decreased parasympathetic nervous system activity [17].

Chronic exposure to trauma and stress can also stimulate the production and release of DAMPs, such as mitochondrial reactive oxygen species (ROS) [57, 58]. DAMPs are key contributors to local or systemic inflammatory responses in the absence of pathogens or tissue damage [57, 58]. Plasma levels of the astroglial protein S100 calcium-binding protein B (S100b), one of the most studied DAMPs in the field of psychiatry, were reported to be higher in veterans with PTSD compared to healthy veterans [59]. Plasma levels of the nuclear protein high mobility group box 1 protein (HMGB1) were increased in severe blunt chest trauma patients with PTSD compared to those without PTSD [60]. In response to stress exposure, these DAMPs bind pattern recognition receptors (PRRs), including the receptor for advanced glycation end-products (RAGE) and toll-like receptors (TLR) on innate immune cells, activating the NF-kB pathway to produce pro-inflammatory cytokines [57, 58].

### Comorbidity between PTSD and immune-related diseases

PTSD can be highly co-morbid with serious physical illnesses, including asthma [61], autoimmune diseases [7, 8], and cardiovascular diseases (CVD) [4, 5]. Multiple studies identified PTSD as a risk factor for CVD and related cardiovascular events, including heart failure and ischemia [62–65], while others propose that CVD symptoms, treatment, and surgery may serve as a trauma that increases PTSD prevalence following acute coronary events [66, 67]. Emerging PTSD symptoms following these cardiovascular events may in turn increase the risk of severe cardiovascular outcomes, including recurrence and mortality [68, 69]. This bidirectional relationship between PTSD and CVD may be due, in part, to contributions from multiple common underlying mechanisms [5, 67, 68], including the ANS [70, 71], HPA axis, oxidative stress [72], and inflammation [5, 73–76].

PTSD is also strongly linked with asthma [3]. The relationship between PTSD and asthma also appears to be bidirectional, as numerous studies report increased asthma prevalence in individuals with PTSD [77–79], while others show a higher odds ratio for PTSD in individuals exhibiting symptoms of asthma [80]. The comorbidity of asthma and PTSD may be explained by shared inflammatory mechanisms. In asthma, the binding of allergens to PRRs triggers both innate and adaptive immune responses [3]. In severe cases of asthma, the T helper 2 (Th2) immune response is augmented by Th17 cells that produce IL-17A, enhancing the pro-inflammatory response [3]. Clinical studies report that those with PTSD do not exhibit differences in Th2 cell proportions [81], but have higher IL-17A levels [82, 83]. Consistent with this finding, those with more severe PTSD symptoms have elevated Th17 cell counts [81]. Overall, this evidence suggests that the link between PTSD and asthma may be driven by an increased Th17 immune response.

PTSD is co-morbid with autoimmune diseases, including inflammatory bowel disease (IBD), rheumatoid arthritis (RA), multiple sclerosis (MS), and psoriasis [7–9]. Notably, the risk of an autoimmune disorder is higher in individuals with PTSD, compared to individuals with other psychiatric disorders [8]. Even though the direction of the association between PTSD and autoimmune diseases is not clear, the fact that this association is not affected by prior trauma and healthy behaviors [7] may suggest that PTSD precedes autoimmune diseases. This hypothesis was supported by a retrospective study of Swedish civilians reporting an increased risk of autoimmune disease development in those with PTSD [84]. Indeed, inflammation is one of the biological mechanisms suspected to link PTSD and autoimmune disorders. Elevated leukocyte, total T-cell counts, and cell-mediated immunity in PTSD may contribute to the development of autoimmune disorders [9, 85, 86].

The inflammatory environment in PTSD may also be exacerbated by co-morbid metabolic conditions [6]. Individuals with PTSD are most likely to suffer from type 2 diabetes mellitus, metabolic syndrome (MetS), and its individual components, including obesity, insulin resistance, and dyslipidemia [8, 87, 88]. This increased comorbidity can be explained by unhealthy lifestyles associated with PTSD (e.g., disrupted sleep patterns, unhealthy diet, tobacco and substance use, physical inactivity), which contribute to inflammation [89–92]. In both MetS and PTSD, the noradrenergic system is activated to trigger an innate immune response [6]. Like PTSD, MetS and obesity are also characterized by an increase in proinflammatory markers, such as CRP, IL-6, and TNF-α [20–22, 93, 94]. Inflammation can promote obesity and insulin resistance, and the resulting fat accumulation, in turn, may lead to elevated levels of proinflammatory cytokines [93, 94]. The connection between PTSD and MetS is supported by a recent hypothesis-free metabolomic study of PTSD that reported dysregulated production and utilization of carbohydrate, lipid, and amino acids, as well as alterations in energy-related pathways [95]. This metabolic evidence indicated inflammation, inefficient energy production, and possibly mitochondrial dysfunction in individuals with PTSD [90]. Mitochondrial dysfunction may lead to increased production of ROS in peripheral organs and immune cells, which contribute to peripheral inflammation. Kusminski and Scherer proposed that inflammation, oxidative stress, and metabolism can be linked together by mitochondrial dysfunction [96]. Overall, a “mitochondrial allostatic load” model may explain the link between these adverse metabolic conditions, inflammation, and PTSD. This model suggests that metabolic dysregulation in PTSD may disrupt mitochondrial activity, resulting in increased ROS production and inflammation [97].

### Other drivers of inflammation in PTSD

Gut microbiota plays an important role in the communication between the brain and the gastrointestinal tract, called the “gut-brain axis”. This axis regulates gastrointestinal homeostasis and links areas of the brain with intestinal functions through the vagus nerve, SNS, and both the endocrine and immune networks [98]. The composition of gut microbiota significantly influences the regulation of the gut-brain axis by stimulating immune cells that contribute to neuroinflammation [99]. Stress, diet, and other environmental factors can disrupt the gut microbiome, which signals the intestinal epithelium to produce pro-inflammatory cytokines [99] and may ultimately lead to permeability in the intestinal tract and excessive antigen trafficking and inflammation [99]. Growing evidence implicates dysregulated gut-brain axis signaling in the pathogenesis of stress and mood disorders and reports gut microbiome alterations in individuals with PTSD [100–103]. Gut microbiome alterations may also mediate the association between early life adversity and symptoms of anxiety in adulthood [104]. These data, in conjunction with evidence showing that PTSD is highly co-morbid with inflammatory gastrointestinal diseases (e.g., IBD), [8, 105] may implicate gut microbiota dysbiosis in the inflammatory environment of PTSD.

Since PTSD disproportionally affects women over men, [106] sex may also modulate the immune response in PTSD [107]. The higher prevalence of PTSD in women can be explained by higher trauma vulnerability, dysregulated fear processing, more sensitive HPA axis, and fluctuating HPA axis activity with the menstrual cycle, as well as aberrant immune responses [17, 106, 107]. Multiple studies reported PTSD-associated differences in immune markers based on sex (reviewed in ref. [108]). A gene co-expression study showed upregulation of an IL-12 signaling module in men but not women with PTSD [31]. Neylan et al. [109] reported increased activation of pathways related to the immune response in monocytes of women, but not men with PTSD. In addition, Kim et al. [110] reported sex-specific differences in peripheral blood leukocyte composition, as men but not women with lifetime PTSD have increased monocyte proportions. Evidence from post-mortem brain samples revealed decreased microglia proportions in women with PTSD [108, 111]. The key factor underlying the sex-specific immune response in PTSD may be estrogen, as studies showed that lower estrogen levels are associated with increased PTSD symptoms [112, 113]. A recent study showed the indirect effect of sex on non-remitting PTSD development through pro-inflammatory cytokines [114]. Authors also showed that this indirect effect of sex was moderated by estradiol, such that men with higher estradiol levels have elevated pro-inflammatory cytokine levels, which was associated with a lower risk of non-remitting PTSD [114]. This relationship can be explained by activation of the HPA axis and inhibition of the SNS by elevated estrogen levels that suppress the production of pro-inflammatory cytokines from T cells and macrophages [107, 115].

Another driver of inflammation in PTSD might be genetics, considering SNP-based heritability estimates of 5–20% that vary based on sex [25]. Supporting evidence comes from studies reporting associations between PTSD and polymorphisms in genes involved in the immune system, including the human leukocyte antigen (HLA) locus [25, 116], *CRP* [117, 118], *TNF-α* [119], and ankyrin repeat domain-55 (*ANKRD55*) [24]. The variations in these immune genes may contribute to pleiotropy between PTSD and immune-related disorders, underlying the shared etiology of these complex and co-morbid disorders. The association between PTSD and the HLA locus is of particular interest, as HLA alleles resulting from combinations of different polymorphisms are heavily implicated in autoimmune disorders (reviewed in [120]). For instance, HLA-A*02:01, which was identified as a protective allele for MS, [121] was less frequent in individuals with PTSD [116], suggesting a plausible shared genetic etiology between PTSD and MS. As HLA alleles have distinct antigen-binding properties, PTSD-associated HLA alleles might have enhanced antigen presentation capacity that impacts T-cell activation and inflammation.

### Impact of inflammation on brain and behavior

**Fig. 3:**
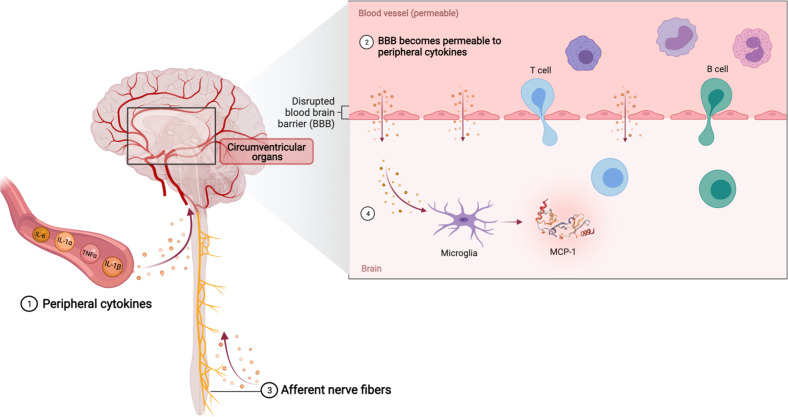
Trafficking of peripheral inflammatory signals to brain. (1) Active transport of peripheral cytokines. (2) Passage of peripheral cytokines through leaky regions of blood–brain barrier (BBB). (3) Transmission of peripheral cytokine signals to the brain by activated cytokine receptors on afferent nerve fibers. (4) Trafficking of peripheral cell types (e.g., monocytes, macrophages, and T cells) in response to monocyte chemoattractant protein (MCP-1) release by activated microglia.

Since PTSD is a brain disorder, there has been a great interest in understanding the mechanisms of neuroinflammation and communication between the brain and the immune system, starting with how peripheral inflammatory responses affect brain function. To date, several mechanisms have been proposed to explain how peripheral inflammatory signals impact the brain (reviewed by [122, 123], Fig. 3): (1) cytokine-specific saturable transporters actively transport some peripheral cytokines (e.g., IL-1α, IL-1β, IL-6, TNF-α), (2) peripheral cytokines pass through the leaky regions in the blood–brain barrier (BBB), (3) activated cytokine receptors on afferent nerve fibers (e.g., vagal nerve) transmit cytokine signals to relevant regions of the brain, and (4) microglial cells that are activated in response to peripheral cytokine signaling produce monocyte chemoattractant protein (MCP-1), which attracts activated peripheral cell types, including monocytes, macrophages and T cells to the brain. Activated microglia and astrocytes also produce cytokines to promote neuroinflammation [18].

Microglia are the resident innate immune-cell type in the brain that are responsible for trophic support, chemotaxis, synaptogenesis, and neurogenesis [124]. Peripheral inflammation activates microglia and prevents microglia from exerting their homeostatic functions [124]. Instead, activated microglia produce pro- and anti-inflammatory cytokines that modulate the stress response in the brain, as noted in animal models of stress and PTSD [125–127]. The inflammatory markers released from microglia induce astrocytes to produce cytokines, which, in turn, activate microglia in a positive feedback loop [128]. Multiple studies in animal models demonstrate microglial activation upon stress through increased pro-inflammatory cytokine response [125, 126, 129] or elevated microglial markers [129, 130]; yet studies of depressed patients and controls showed no correlations between microglial activation and peripheral pro-inflammatory cytokine levels [131, 132]. In contrast, a recent study reported lower microglial activation in post-mortem brain samples of PTSD patients [133]. The authors also demonstrated a negative correlation between microglial activation and plasma CRP levels [133]. The inconsistencies in stress-induced microglial activation might be due to differences between species, stress type, or analysis strategies to assess microglial activation (e.g., immunohistochemistry vs. neuroimaging).

#### Effect of inflammation on neurocircuitry relevant to fear and anxiety

PTSD is associated with alterations in the brain regions relevant to fear, anxiety, and threat detection, such as the amygdala, hippocampus, medial prefrontal cortex (mPFC), anterior cingulate cortex (ACC), and insula [17, 123]. Hence, evaluating the effect of inflammation on these regions is relevant to understanding the behavioral changes associated with PTSD.

The amygdala is the brain region responsible for fear and anxiety responses and is hyperresponsive in individuals with PTSD [123]. Multiple neuroimaging studies reported an association between heightened amygdala activation upon stress and increased proinflammatory cytokine levels. For instance, increased IL-6 and TNF-α concentrations following endotoxin administration in healthy individuals resulted in increased amygdala activity in response to socially threatening images [134]. Increased amygdala response to congruent and incongruent stimuli was also associated with increased IL-6 levels upon vaccination [135]. Notably, increased pro-inflammatory cytokine levels and amygdala activation are also associated with social disconnection, depressed mood, cognitive disturbance, and fatigue [135, 136].

The hippocampus is involved in fear and memory processing [137, 138]. Importantly, individuals with PTSD have smaller hippocampal volume [139]. In addition, reduced hippocampal volumes are associated with increased inflammation [140]. The effect of inflammation on the hippocampus was assessed in rodent models [141, 142], which suggests that microglial release of cytokines suppresses neurogenesis and stimulates apoptosis of neuronal progenitor cells [143]. Studies also showed the inhibitory effect of IL-1β on long-term potentiation in the hippocampus [144], as well as spatial and contextual memory processing [145]. Hence detrimental effects of inflammation on the hippocampus may be an underlying contributor to cognitive and emotional problems associated with PTSD.

Through their connections to the amygdala and hippocampus, the mPFC regions, including the rostral ACC, subgenual ACC (sgACC, Brodmann’s Area 25), and the medial frontal cortex, play an important role in emotional regulation and fear extinction in PTSD [17]. Multiple studies investigated the effect of peripheral inflammatory markers on mPFC activity in response to stress or upon cytokine inducement [146, 147]. For instance, activation of the ventral mPFC, including the sgACC and the orbitofrontal cortex (OFC), in response to a grief-elicitation task associated with elevated IL-1β and sTNF-RII levels in grieving women [147]. Likewise, elevated IL-6 levels following typhoid vaccination led to increased sgACC activity, which is correlated with mood deterioration, and decreased connectivity of the sACC to the amygdala and mPFC [146]. In addition, increased plasma levels of CRP and IL-6 were correlated with reduced connectivity between the striatum and the ventral mPFC in depressed patients [148]. Finally, exposure to an acute laboratory-based social stressor led to an increase in IL-6 levels, which was associated with stronger functional connectivity between the right amygdala and the dorsomedial PFC [136]. Overall, this evidence links mPFC activation and inflammation in emotion processing following trauma or stress.

The dorsal ACC (dACC, Brodmann’s Area 24) is involved in emotional and physical stress response through threatening social and physical pain stimuli detection and response [123]. Hyperactivation of the dACC is associated with PTSD [137, 149–152] and has been shown to mediate hyperarousal symptoms of PTSD [153]. Neuroimaging studies also demonstrated activation in response to inflammation. For example, IFN-α treatment of hepatitis C patients led to heightened dACC activation, which is associated with visual–spatial-attention errors [154]. Similarly, elevated IL-6 concentrations following typhoid injection are associated with increased dACC activation [135]. Finally, increased IL-6 levels upon endotoxin administration have been shown to be associated with augmented neural activity related to social pain in the dACC of women [155], consistent with sex-specific immune responses in PTSD. Taken together, these data suggest that dACC hyperactivation in response to inflammation may underlie some of the behavioral changes observed in PTSD.

The insula is involved in the emotional distress symptoms of PTSD and plays an important role in interoception (i.e., sense of body’s physiological state) [123]. The insula is activated by peripheral inflammatory stimuli, which is expected considering the role of this region in perceiving the signals from the body [135]. Lower insula activation in response to changes in interceptive responses associated with PTSD in women exposed to intimate partner violence [156]. Likewise, women with PTSD related to intimate partner violence exhibited heightened activation of the insula and the amygdala, as well as weaker functional coupling among the insula, amygdala, and ACC during an emotional face-matching task [157]. Notably, the insula is also a target of the peripheral inflammatory response, such that IL-6 increases following stimulation of innate immunity led to heightened insula activity in response to congruent and incongruent stimuli [135]. Moreover, increased IL-6 and TNF-α levels following endotoxin administration are associated with increased glucose metabolism in the insula, as well as behavioral changes, including fatigue and lower social interest [158]. Similarly, increased IL-6 levels are associated with higher insula activity in response to social pain in women [159]. Hence, pro-inflammatory cytokines may lead to insula hyperactivity and alter the neural circuitry of the amygdala, mPFC, and ACC, thereby contributing to PTSD symptomatology relevant to fear and emotion processing.

#### Possible mechanism by which inflammation alters neurotransmitter function

Cytokines are critical to maintaining neural homeostasis, by participating in neural plasticity, including neurogenesis, synaptic pruning and remodeling, long-term potentiation, learning, and memory [145]. However, increased inflammatory signaling has detrimental effects on neurotransmitter systems related to the behavior and emotional characteristics of PTSD, such as serotonin, norepinephrine, dopamine, and glutamate [17, 122]. Inflammatory cytokines can alter neurotransmitter functions by influencing synthesis, reuptake, and release of neurotransmitters [122].

Serotonin is a monoamine neurotransmitter that is widely implicated in the etiology and pathophysiology of PTSD [160]. Serotonergic signaling may be influenced by the immune system in PTSD. Rats immunized with *Mycobacterium vaccae*, which exerts immunoregulatory properties through the production of anti-inflammatory cytokines, showed enhanced fear extinction and altered serotonergic gene expression in the brainstem [161, 162].

**Fig. 4:**
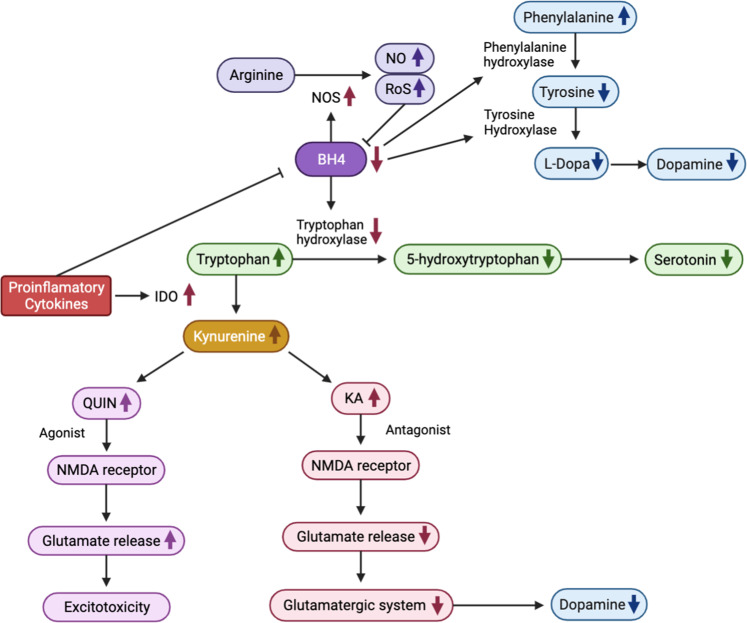
Pro-inflammatory cytokine-induced changes in neurotransmitter systems. Mechanisms by which proinflammatory cytokines affect the synthesis of monoamine neurotransmitters (i.e., serotonin and dopamine) are illustrated. BH4 tetrahydrobiopterin, IDO indoleamine 2,3-dioxygenase, KA kynurenic acid, NMDA N-methyl-d-aspartate, NO nitric oxide, NOS nitric oxide synthases, QUIN quinolinic acid, ROS reactive oxygen species.

Cytokines can influence serotonin synthesis through the kynurenine pathway (Fig. 4). Pro-inflammatory cytokines increase the activity of indoleamine 2,3-dioxygenase (IDO), which converts tryptophan, the primary amino acid of serotonin, into kynurenine [122]. Hence, increased IDO activity in response to inflammation leads to serotonin depletion in the brain, as observed in animal models [163, 164]. In human studies, IFN-α therapy led to increased kynurenine and decreased tryptophan levels that were associated with symptoms of depression and anxiety, as well as cognitive problems, including memory disturbances and confusion [165, 166]. Another study of IFN-α therapy reported higher TNF-α and lower serotonin concentrations associated with somatic symptoms, including fatigue, loss of appetite, and irritability [167]. These findings suggest that pro-inflammatory cytokines may reduce serotonin concentration by acting on the kynurenine pathway, leading to cognitive and somatic symptoms relevant to PTSD.

Cytokines also downregulate serotonin synthesis by decreasing the activity of tetrahydrobiopterin (BH4), an enzyme co-factor of tryptophan hydroxylase and a rate-limiting enzyme in serotonin synthesis [122] (Fig. 4). Cytokines can increase the expression and function of serotonin transporters by stimulating the p38 mitogen-activated protein kinase MAPK pathway. Several in vitro studies have shown increased expression and activity of the serotonin transporter through activation of MAPK following TNF-α and IL-1β stimulation [168, 169]. Importantly, fluoxetine, a selective serotonin reuptake inhibitor (SSRI), suppressed the expression of IL-1β, IFN-γ, and TNF-α in the rat hippocampal dentate gyrus and downregulated MAPK signaling [170].

Dopamine plays an important role in PTSD symptom clusters, including re-experiencing symptoms and negative mood and cognition [171]. Cytokines reduce dopamine levels by diminishing the activity of BH4, a co-factor of tyrosine hydroxylase and phenylalanine hydroxylase, the rate-limiting enzymes for dopamine synthesis [122] (Fig. 4). Inflammation can also induce nitric oxide synthase (NOS), the enzyme responsible for converting arginine into nitric oxide (NO), which uses BH4 as a co-factor [122]. Depletion of BH4 in turn leads to NOS uncoupling and production of ROS in the brain. Since BH4 is highly sensitive to oxidative stress, ROS promotes irreversible degradation of BH4, further limiting BH4 availability [123]. In fact, treatment of sympathetic neurons with IL-6 led to lower BH4 levels [172]. Similarly, IFN-α-treated patients exhibited decreased BH4 activity [173, 174], which was associated with lower dopamine levels in their cerebrospinal fluid (CSF) [173].

Glutamate is involved in motivation and motor functions. Inflammation leads to increased glutamate levels, contributing to symptoms of emotional numbness in PTSD [123]. The glutamatergic system partly regulates dopamine release, such that the effect of cytokines on the kynurenine pathway also impacts dopamine synthesis. As discussed above (Fig. 4), cytokines enhance IDO activity, leading to an increase in kynurenine production. Kynurenine can be broken down to kynurenic acid (KA) in astrocytes and quinolinic acid (QUIN) in microglia [123]. Notably, patients undergoing IFN-α treatment showed increased KA and QUIN concentrations in plasma and CSF [122, 123]. KA is an N-methyl-D-aspartate (NMDA) receptor antagonist that inhibits glutamate release and has downstream effects on dopamine [175, 176]. QUIN, an N-NMDA receptor agonist, can activate glutamate release from astrocytes, thereby contributing to excitotoxicity in the brain [123, 175]. Patients undergoing IFN-α treatment showed increased glutamate to creatinine levels in the dACC, which was associated with depressive symptoms [177]. Elevated CRP levels associated with symptoms of depression led to increased basal ganglia glutamate levels [178]. The increased glutamate release from astrocytes reduced brain-derived neurotrophic factor (BDNF), which is essential for neurogenesis and associated with disrupted contextual fear memory in PTSD [179, 180].

The effect of inflammation on other neurotransmitter systems related to PTSD, such as gamma-aminobutyric acid (GABA) and acetylcholine was less studied. PTSD patients were shown to have lower GABA levels in insula [181]. Notably, GABA decreased the production of inflammatory cytokines by suppressing the NF-kB and p38 MAPK pathways in rodents [182]. In addition, the expression and activity of acetylcholinesterase were induced by proinflammatory cytokines, which inhibited the release of acetylcholine from hippocampal neurons [183, 184]. Inhibition of acetylcholine release may, in turn, contribute to inflammation, as acetylcholine can downregulate peripheral cytokine production via the “cholinergic anti-inflammatory reflex.” [122]. These data suggest that cytokines may reduce the release of GABA and acetylcholine, which both have anti-inflammatory effects, thereby leading to an inflammatory environment.

Taken together, studies collectively suggest that trauma may lead to HPA axis and SNS activation that increases proinflammatory cytokine production and subsequent neurotransmitter signaling that increases the risk of fear and anxiety symptomatology. Ultimately, this cascade may contribute to the risk of PTSD onset.

## Potential anti-inflammatory therapeutic approaches

Currently, only two selective SSRIs, paroxetine and sertraline, are approved by the FDA for the treatment of PTSD [18]. However, the response rates of these SSRIs are lower than 65% [185–188], indicating that a large proportion of PTSD patients do not respond to SSRI treatment. Hence, given the inflammatory characteristic of PTSD, strategies that reduce inflammation and/or its effects on the brain may provide new avenues for the development of curative or preventative treatments to be used as an adjuvant to SSRIs or in combination with behavioral approaches.

Monoclonal antibodies to cytokines and their receptors, including TNF, IL-1, IL-6R, IL-12/23, and IL-17, are approved by the FDA for the treatment of autoimmune diseases and cancers. Given the increased IL-1β, IL-6, and TNF-α levels in PTSD, blocking these cytokines may be a straightforward treatment strategy. Although there are no reports of monoclonal antibody use for the treatment of PTSD, multiple studies reported that etanercept (TNF inhibitor), adalimumab (TNF inhibitor), and ustekinumab (IL-12/23 inhibitor) reduced symptoms of depression and anxiety in individuals with psoriasis [189–192]. However, Raison et al. [193] showed that infliximab (TNF inhibitor) was only effective in treatment-resistant depressed patients with higher baseline inflammation. Further, a clinical trial in patients with bipolar depression reported that baseline inflammation moderated the effect of infliximab on reducing anhedonia symptoms [194].

Non-steroidal anti-inflammatory drugs (NSAIDs) and cyclooxygenase 2 (COX-2) inhibitors negatively regulate proinflammatory cytokine production, and thus reduce inflammation. The COX-2 inhibitor celecoxib was shown to improve depression symptoms in patients with the major depressive disorder [195–197], potentially decreasing IL-6 levels [197]. Although no clinical studies evaluated NSAIDs and COX-2 inhibitors for the treatment of PTSD, COX-2 inhibitors were shown to reduce anxiety in mice exposed to stress [198]. In addition, ibuprofen (NSAID) treatment reduced anxiety symptoms in a rat model of PTSD while decreasing expression of *TNF-α* and *IL-1β* and increasing *BDNF* expression in the hippocampus, suggesting that the therapeutic effect of ibuprofen on PTSD was mediated by decreased anti-inflammatory activity and increased BDNF levels in the brain [199].

NACHT domain- leucine-rich repeat- and pyrin domain-containing protein 3 (NLRP3) inflammasome inhibitors block inflammatory cytokine production. Beta-hydroxybutyrate (BHB), an endogenic NLRP3 inflammasome inhibitor, was shown to reduce depressive and anxiety behaviors in rodent models of depression and stress, potentially though decreasing hippocampal TNF-α concentrations [200, 201]. BHB was also effective in reducing anxiety behaviors in rodent models of PTSD and restoring serum TNF-α levels that were elevated in response to single prolonged stress [202].

Glucocorticoids suppress the inflammatory response following stress exposure by promoting the production of anti-inflammatory cytokines and by inhibiting the synthesis of proinflammatory cytokines [18] (Fig. 2). In addition, glucocorticoids participate in transporting protective T cells to the brain during acute trauma [203]. Given the low cortisol concentrations in individuals with PTSD [18], clinical studies examined the effectiveness of synthetic glucocorticoids for treatment of PTSD or preventing PTSD development following acute traumatic stress. Clinical trials showed that stand-alone glucocorticoid treatment or glucocorticoid augmentation combined with psychotherapy improved PTSD symptoms [204–207]. However, a recent clinical trial testing the effectiveness of augmentation of prolonged exposure (PE) with glucocorticoid reported that glucocorticoid augmentation did not significantly ameliorate PTSD symptoms [208]. Still, their exploratory analyses showed that glucocorticoid augmentation improved hyperarousal symptoms in veterans who experienced mild traumatic brain injury and reduced avoidance symptoms in veterans with increased baseline glucocorticoid sensitivity [208]. Moreover, studies investigating the preventative effect of glucocorticoids following exposure to trauma showed that glucocorticoid treatment following acute trauma significantly reduced stress symptoms [209, 210] and decreased the incidence of PTSD [211, 212]. Recently, a large meta-analysis of randomized controlled trials of glucocorticoid treatment reported that, although glucocorticoid treatment alleviated PTSD symptoms, preventative glucocorticoid administration following acute trauma was more effective [213].

Noradrenergic beta-receptor blockers (e.g., propranolol) inhibit norepinephrine signaling that promotes the production of pro-inflammatory cytokines [53, 54]. Studies of animal models showed that propranolol administration following stress exposure reduced pro-inflammatory cytokine levels and abrogated stress-induced changes in the immune-cell composition [214, 215]. Importantly, blockage of noradrenergic beta-receptors has been shown to inhibit reconsolidation of fear memory [216]. Indeed, clinical trials reported that propanol treatment with memory reactivation therapy reduced PTSD symptoms [217, 218]. Beta-blocker administration for the suspected acute coronary syndrome was also shown to reduce PTSD symptoms at 1-month follow-up [219]. However, research on the preventative effects of propranolol is contradictory. Initial studies reported that propanol treatment initiated within 20 hours of the trauma lowered PTSD incidence 2-months after trauma exposure and reduced physiological reactivity to trauma cues after 3 months [220, 221]. In contrast, Stein et al. [222] reported that propanol administration within 48 hours of trauma had no benefits on PTSD symptoms at 1, 4, and 8 months follow-up. Since propanol appears to act by reducing the effect of SNS arousal on trauma memory consolidation, early initiation of propanol treatment seems to be crucial.

Angiotensin-converting enzyme inhibitors (ACE-I) and angiotensin receptor blockers (ARBs) have been evaluated as possible anti-inflammatory therapeutic strategies, considering they are effective in the treatment of cardiometabolic disorders that are highly co-morbid with PTSD [17]. ACE-I and ARBs exert their anti-inflammatory activity in the brain by reducing the expression and secretion of pro-inflammatory cytokines and decreasing microglial activation [223]. Candesartan (ACE-I) was shown to ameliorate impairment of fear extinction in response to inflammatory activity induced by lipopolysaccharide administration [224]. A cross-sectional clinical observation study reported that individuals with PTSD on ACE-I or ARB treatment have lower hyperarousal symptoms compared to patients not taking these medications [225]. This study also showed that ACE-I or ARB use was associated with lower PTSD symptoms in trauma-exposed individuals [225]. However, a recent clinical trial reported that losartan (ARB) did not ameliorate PTSD symptoms [226].

Cannabinoids are also considered for PTSD treatment due to their anti-inflammatory effects. Endocannabinoid (eCB) signaling from macrophage and monocyte cells, including microglia in the brain, participates in inflammatory processes related to PTSD. While deficient eCB signaling promotes inflammation, augmented eCB signaling suppresses inflammation by reducing the secretion of pro-inflammatory cytokines, inhibiting NF-kB-mediated inflammatory gene transcription, decreasing microglial activation, and promoting the release of anti-inflammatory cytokines [227, 228]. Multiple studies showed that nabilone, a synthetic cannabinoid, reduced PTSD-related nightmares and insomnia and improved PTSD symptoms [229–231].

FTY720 (Fingolimod), approved for the treatment of MS, has drawn researchers’ interest for PTSD treatment. Fingolimod is a synthetic analog of sphingosine that non-selectively binds to sphingosine-1-phosphate receptors (S1PRs). Decreased S1PRs activity in response to fingolimod prevents leukocyte migration from lymphocytes to the CNS, thereby suppressing the immune response [232]. Fingolimod was shown to promote neurogenesis, which correlated with improved contextual fear memory in mouse models [233, 234]. Notably, Fingolimod decreased despair and social anxiety-like behavior and reduced blood lymphocyte counts in a rat model of stress by reducing vascular remodeling in the brain [235].

Psychotherapy and behavioral interventions are effective treatments for PTSD and may reduce inflammation by reducing perceived stress and increasing emotion regulation [236]. Currently, there are no studies evaluating the effect of gold-standard PTSD psychotherapies, including PE, on inflammation. Nevertheless, other forms of psychotherapy, including eye movement desensitization, were associated with alterations in TNF-α in soldiers with PTSD [237]. A recent clinical trial of reminder-focused positive psychiatry on attention-deficit hyperactive disorder and PTSD reported a decrease in CRP levels at 6 weeks follow-up [238]. Moreover, other behavioral interventions, including yoga and mindfulness, are associated with decreases in inflammation [236, 239].

## Concluding remarks

A growing body of evidence indicates an inflammatory environment in individuals with PTSD. However, many of the epidemiologic studies were limited by the fact that they only investigated specific peripheral cytokines and potentially missed key regulators in the process. Animal models deficient in key inflammatory genes may help identify mechanisms underlying the relationship between inflammation and anxiety-like or social behaviors relevant to PTSD. Similarly, longitudinal epidemiologic studies will be necessary to understand the directionality between PTSD and inflammation as well as immune-related conditions co-morbid with PTSD. For instance, since the gut-brain axis plays an important role in both PTSD and IBD, microbiome studies of PTSD coupled with metabolomics may advance our understanding of this comorbidity.

It is also unclear how peripheral immune alterations are indicative of neuroinflammation. Findings from imaging studies support a link between peripheral inflammation and both cognitive and emotional problems associated with PTSD through alterations in neurocircuitry relevant to fear and anxiety (reviewed in [123]). While this is encouraging, many of the reports of changes in neurotransmitter function in response to inflammation were from studies of depression, and additional research targeting PTSD is required to fully understand how the alterations in neurotransmitter function are relevant to PTSD symptoms.

Finally, the link between PTSD, inflammation, and the brain paves the way for potential anti-inflammatory treatment or preventative therapies. Although multiple anti-inflammatory treatment strategies showed promising results in pre-clinical settings and clinical trials, clinical studies with larger sample sizes and more diverse populations are warranted to fully understand the therapeutic mechanism of action, effectiveness, and possible side effects of these anti-inflammatory treatments.
